# Postsystolic Shortening Is Associated with Altered Right Ventricular Function in Children after Tetralogy of Fallot Surgical Repair

**DOI:** 10.1371/journal.pone.0169178

**Published:** 2017-01-03

**Authors:** Radosław Pietrzak, Bożena Werner

**Affiliations:** Department of Pediatric Cardiology and General Pediatrics. Medical University of Warsaw Zwirki i Wigury, Warsaw, Poland; Universitat de Barcelona, SPAIN

## Abstract

The aim of the study was to determine whether segmental interactions, as expressed by postsystolic shortening (PSS), affects RV mechanics and are connected with impaired systolic and diastolic function in rTOF children. Patients and Methods: 55 rTOF adolescent (study group), and 34 healthy volunteers (control group) were examined using classical Doppler flow (Doppler), Tissue Doppler Imaging (TDI) and Speckle Tracking Echocardiography (STE). PSS was found to occur when time to peak (TTP) was longer than pulmonary valve closure time (PVCT). TTP and strain were derived from RV lateral segments—basal (BL), medial (ML) and apical (AL) in STE. PVCT was measured from the beginning of QRS complex in the ECG to the termination of Doppler flow at the pulmonary valve. TDI was obtained at the lateral tricuspid annulus site and the systolic (S′), early (E′) and late diastolic (A’) peak velocities were measured along with isovolumic contraction (IVCT), and relaxation (IVRT) time. PW was used to measure early tricuspid inflow velocity (E) for calculating the E/E’ ratio. The TDI data in patients with PSS presence (TTP>PVCT) and those in whom it did not occur (TTP≤PVCT) were compared. Results: PSS in BL, ML and AL were observed respectively in: 27(51,9%), 9 (18%), and 8 (16,7%) patients. Mean values of TTP in BL, ML, and AL were respectively: 420.6±55.5ms, 389.8±50.0ms and 366.7±59.0ms. PVCT mean value was 396.6±33.5ms. In the study group, the mean E’ in TTP>PVCT was significantly lower (4.8±1.8 cm/s) compared to mean E’ in TTP≤PVCT (8.4±2.6 cm/s), p<0.01. The average E/E’ was significantly higher in TTP>PCVT than in TTP≤PVCT, respectively 21.6±7.3 vs 12.2±5.1, p<0.05. IVRT was significantly prolonged in TTP>PVCT compared to IVRT in TTP≤PVCT, respectively 95.9±38.7 vs 77.0±35.1, p<0.05. Furthermore, in TTP>PVCT, significantly higher strain in BL (-28.8±8.7%) was observed when compared to that parameter in TTP≤PVCT (-35.3±13.1%), p <0.05. Conclusions: Tissue Doppler Echocardiography and Speckle Tracking Echocardiography are useful techniques for detecting regional systolic and diastolic dysfunction in children after Tetralogy of Fallot surgical repair. Postsystolic shortening in the basal lateral segment is commonly seen in children after the Tetralogy of Fallot surgical repair, and is associated with altered right ventricular systolic and diastolic function.

## Introduction

An increasing number of patients after Tetralogy of Fallot surgical repair (rTOF) has led to a growing adolescent population with postoperative cardiac sequels. [1] Pulmonary regurgitation has been observed to be a major residual lesion associated with right ventricular (RV) volume overload, driving towards its dysfunction in rTOF patients. [2,3] Furthermore, the almost invariable presence of right bundle branch block (RBBB) in rTOF patients may play an important role in their long-term clinical outcomes, where it is likely that electrical and mechanical dysfunction are linked. [4,5] For the latter, RV abnormalities occur during both systole and diastole, with global and regional abnormalities in systole being well documented [6,7]. The diastolic dysfunction is however less known. Previous studies have focused on markers of decreased late diastolic myocardial compliance, reflected by restrictive physiology of the RV [8]. Nonetheless, some trials have demonstrated the postsystolic shortening phenomenon (PSS) to be a mechanism for early diastolic dysfunction in left-sided lesions [9–12]. Only one trial however shows a PSS within the RV, where an elegant model of right ventricular lateral wall activation was presented [13]. For this sequence early septal activation, as observed echocardiographically by a septal flash with concomitant lateral wall prestretch, follows contraction of the early stretched lateral wall. The subsequent late initiation of systolic RV lateral contraction (connected with RBBB) may then be partially inefficient as it continues on after pulmonary valve closure. Another postulated mechanism responsible for the PSS phenomenon is passive post-systolic deformation after end-systolic stretch observed at the pulmonary/aortic valve closure [14,15].

We thus hypothesized, those segmental interactions, as expressed by postsystolic shortening, affects RV mechanics and are connected with impaired systolic and diastolic function in rTOF children.

## Patients and Methods

Study subjects were 55 consecutive rTOF patients, aged 8–18 years (average 13.7±3.3 years). The control group was comprised of 34 healthy volunteers aged 8–18 years, (average 13.6±2.9 years). The data on surgical treatment in the study group were obtained from medical documentation. Anthropometrical parameters were measured in the study and control group.

In all patients, resting ECG was performed to evaluate the shape and duration of the QRS complex.

Echocardiography: patients and controls were examined using a standardized protocol by the iE 33 ultrasound system (Philips); being in the left lateral decubitus position with a phased-array transducer S5-1. The RV focused apical 4-chamber view (RV AP4Ch) was used to calculate end-systolic (ESA) and diastolic (EDA) areas. These were adjusted to body surface area. The right ventricular fractional area change (RVFAC) was calculated by: EDA-ESA/EDA x100%. RV AP4Ch was also used to assess transannular plane systolic excursion in M-mode. Classical Doppler flow was obtained at the pulmonary valve and tricuspid valve. Tissue Doppler Imaging (TDI) was measured at the lateral tricuspid annulus site at a frame rate of at least 90 Hz from the RV AP4Ch. Gray-Scale images for RV speckle tracking echocardiography (STE) were acquired at frame rates of 50 to 90 Hz also from the RV AP4Ch. At least 5 cardiac cycles were stored for offline analysis (“QLAB Advanced Quantification” Philips). Tracking was automatically generated by the software and was only accepted if visual inspection indicated adequate covering of the myocardium throughout the whole cardiac cycle. Strain curves were generated at the basal, mid, and apical RV lateral wall. Patients in whom STE was inadequate in more than 1 segment were excluded.

The forward pulmonary flow and pulmonary regurgitation (PR) were assessed in continuous wave Doppler. The maximal and mean systolic pressure gradient between the right ventricular outflow tract and main pulmonary artery were measured. The ratio between the duration of PR and total diastole, referred to as PR index, was used to assess the degree of PR as previously described in the literature [16]

The tricuspid valve flow and regurgitation was assessed in the apical 4-chamber view. Pulse wave Doppler was used to measure a maximal early (E) and atrial (A) tricuspid inflow velocity along with E deceleration time. The E/A ratio was calculated. Tricuspid regurgitation was assessed in color Doppler flow and was considered significant when the ratio of regurgitant jet area to total right atrial area was at least 40% with an optimal setting of color Doppler gain and, additionally, when evidence of retrograde flow in the inferior vena cava in PW was registered.

TDI was obtained at the lateral tricuspid annulus site and the systolic (S′), early diastolic (E′) and late diastolic (A’) peak velocities were measured along with isovolumic contraction (IVCT) and relaxation (IVRT) time. The E/E’ ratio was thence calculated

**Fig 1:**
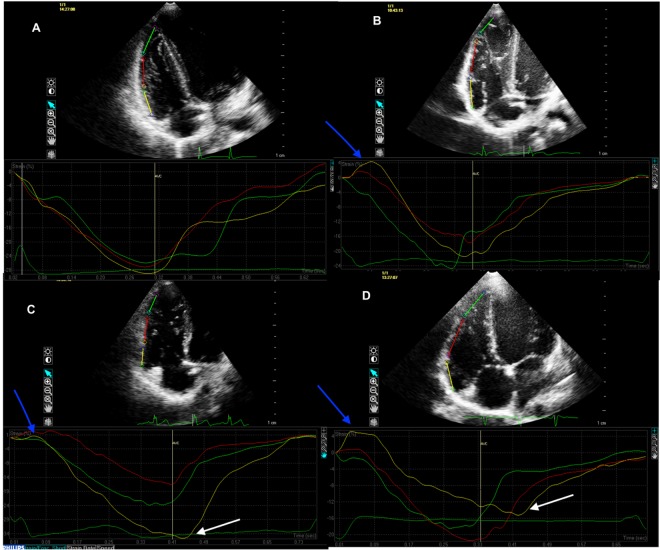
Speckle Tracking Echocardiography. Apical 4-chamber view. Postsystolic shortening. A. control, B study patient; prestretch present (+), PSS not present (-), C. study patient; prestretch (+), PSS(+) D. study patient; prestretch (+), PSS(+), deformation in BL lower than in ML. white arrow–postsystolic shortening, blue arrow–presystolic stretch, AVC–pulmonary valve closure.

In STE, time to peak (TTP) and maximal value of longitudinal strain (ε) were automatically derived from each of the three RV segments. The early prestretch, defined as a segmental elongation (stretch) before shortening, was recorded. PSS was found to occur when the TTP was longer than the pulmonary valve closure time (PVCT). PVCT was measured from the beginning of QRS complex in the ECG to the termination of flow at the pulmonary valve in pulse wave Doppler flow. The duration of deformation after pulmonary valve closure, i.e. the postsystolic shortening time (PSST), was calculated by: PSST = TTP–PVCT, whilst the postsystolic shortening time index (PSSTi), was obtained by: PSSTi = PSST/PVCP x 100%, where PVCP is the pulmonary valve closure period. PVCP was defined as the time between termination and beginning of pulmonary flow in pulse wave Doppler. Strain at the moment of pulmonary valve closure (εPVC) was measured manually and postsystolic shortening strain was calculated, (i.e. the εPSS; the extent of deformation after pulmonary valve closure), using the formula: εPSS = ε—εPVC. The postsystolic shortening strain index (εPSSi) was also determined by: εPSSi = εPSS/ ε. Fig 1 presents various patterns of strain within the right ventricular free wall.

ECG was registered during the whole examination. Maximal accepted difference in mean values of RR intervals between the ECG’s in the 2-dimensional images and the Pulse Wave Doppler was 5%. The study received approval from the Bioethics Committee, Medical University of Warsaw No: KB/18/2009. All study subjects’ parents/guardians signed written informed consent form; additionally the study participants aged above 15 years old also signed written informed consent form. The Bioethics Committee, Medical University of Warsaw, approved all these documents.

All data underlying the findings described in our manuscript are fully available as supporting information files.

### Statistical analysis

The numbers and rates of patients and healthy children with and without postsystolic shortening were counted. Data were presented as a mean ± SD. The Shapiro-Wilk test was used to verify a normal distribution. Comparison between the controls and the study group were determined by using the Mann-Whitney U test. 2x2 tables were analyzed using the exact Fisher test. Correlations were determined according to the Spermann rank order correlation. The statistical analysis was not performed if the number of patients was less than 10. A p<0.05 was taken as being statistically significant.

## Results

The mean patient age at the time of surgical correction was 12±8.9 months. The trial was performed 12.9±3.3 years after reparative surgery. Three patients of the study group (none of the controls) were excluded from the trial, when tracing was inadequate in more than 1 segment. For the further analysis 52 patients were considered. Eleven (21.1%) patients received a transannular patch during initial surgical repair. Nine patients (17.3%) underwent Blalock-Taussig shunt before corrective surgery whilst one (1.9%) underwent reoperation for a residual ventricular septal defect. Both study and control groups had statistically similar body mass, height and body surface area (Table 1).

**Table 1:** Anthropometric parameters in the study and control group.

	Study Group,mean ± SD	Control Group, mean ± SD	p
Height [cm]	159.4±16.8	157.6±16.3	NS
Weight [kg]	52.3±15.6	50.8±16.1	NS
Body surface [m^2^]	1.5±0.3	1.5±0.3	NS

The complete right bundle branch block was found in 48 (92%) children of the study group and none for children in the control group. The average value of the QRS duration was statistically significantly longer (p<0.01) in patients after the tetralogy of Fallot correction (value 137±21 ms) compared with QRS duration in the control group (value 77±12 ms).

In study group patients, severe pulmonary regurgitation was seen in 17(32.7%), moderate in 24 (46.1%), and mild in 11 (21.1%). There was no diagnosis of moderate and severe pulmonary regurgitation in healthy children. Three (3.6%) of the study patients had severe tricuspid valve regurgitation but none in healthy volunteers.

Results of the remaining of echocardiographic parameters in the control and study group are summarized in Table 2.

**Table 2:** Echocardiography in the study and control group.

		Study Group,mean ± SD	Control Group,mean ± SD	p
RV ESA/BSA [mm^2^/m^2^]		13.7±6.2	6,1±3.0	<0.01
RV EDA/BSA [mm^2^/m^2^]		19.2±7.8	11.9±4.8	<0.01
TAPSE [mm]		16.5±4.0	19.4±3.2	<0.01
RVFAC [%]		28.8±8.1	49.4±10.1	<0.01
PVCP [ms]		391.8±114.6	398.8±56.4	NS
Max PG [mmHg]		23.1±13.3	7.3±2.4	<0.01
Mean PG [mmHg]		12.3±7.5	3.2±1.1	<0.01
E [cm/s]		91.5±15.7	61.7±13.5	<0.01
A [cm/s]		67.6±16.2	45.6±12.2	<0.01
Edt [ms]		190.7±51.2	192.2±38.0	NS
E/A		1.4±1.3	1.4±0.3	NS
S’ [cm/s]		5.7±1.6	11.0±2.3	<0.01
IVCT [ms]		117.0±59.6	60.9±25.8	<0.01
E’ [cm/s]		6.5±2.8	16.2±3.4	<0.01
A’ [cm/s]		3.8±1.5	7.8±2.0	<0.01
IVRT [ms]		86.8±36.7	40.8±13.9	<0.01
E/E’		17.2±8.5	4.1±1.4	<0.01
BL	ε[%]	-32.6±11.6	-48.6±15.5	<0.01
	TTP[ms]	420.6±55.5	368.8±41.7	<0.01
ML	ε[%]	-23.1±8.9	-39.5±14.8	<0.01
	TTP [ms]	385.3±47.6	360.3±43.8	<0.05
AL	ε[%]	-16.6±7.8	-36.2±12.9	<0.01
	TTP[ms]	367.2±56.6	362.2±67.4	NS

TAPSE–transannular plane systolic excursion, RV FAC–right ventricular fractional area change, RV ESA/BSA–right ventricular end systolic area indexed to body surface area, RVEDA/BSA right ventricular end diastolic area indexed to body surface area, PVCP–pulmonary valve closure period, max PG—maximal systolic pressure gradient between right ventricular outflow tract and pulmonary artery, mean PG- mean systolic pressure gradient between right ventricular outflow tract and pulmonary artery, E—maximal early tricuspid inflow velocity, A—atrial tricuspid inflow velocity, Edt–E deceleration time, E/A–E/A ratio, S’–peak systolic velocity of the tricuspid valve, ICT—isovolumic contraction time, E’—peak early diastolic velocity, A’ peak late diastolic velocity, IVRT- isovolumic relaxation time, E/E’—E/E’ ratio, ε–maximal value of longitudinal strain, TTP–time to peak, BL–basal lateral segment, ML–medial lateral segment, AL–apical lateral segment.

The early prestretch was seen in 49 (94.2%), 12 (23%), 7(13%) patients of the study group and in 3 (8%), 2 (5,8%) and none of healthy children in basal, medial and apical lateral segments respectively. PSS occurred also more frequently in the study group than controls for all segments measured in the right ventricle (Table 3). Patients in whom postsystolic shortening in basal lateral segment was seen were PSS+ and those in whom it was not seen were PSS-. Because of the low numbers of children, further analysis of the mid and apical segments, were therefore not performed. Early prestreach in BL was seen in 26 (96%) PSS+ patients and in 23 (92%) PSS- patients.

**Table 3:** Postsystolic shortening in lateral free wall of the right ventricle.

	Study group, number of patients (percentage)	Control group, number of patients (percentage)	p
Basal lateral segment—BL			
PSS+	27(51.9%)	10(29.4%)	NS
PSS-	25(48.1%)	24(70.5%)	
Medial lateral segment—ML			
PSS+	9(18.0%)	5(14.7%)	*
PSS-	41(82.0%)	29(85.3%)	
Apical lateral segment—AL			
PSS+	8(16.7%)	4(11.8%)	*
PSS-	40(83.3%)	30(88.2%)	

PSS+ post systolic shortening present. PSS- post systolic shortening absent.

* further analysis not performed due to low numbers of patients.

### PSS + and PSS—patients

The mean value of maximum E’ wave velocity was significantly lower in PSS+ patients compared to PSS- children from the study group p<0.01. The average E/E’ value was significantly higher in PSS+ than in PSS- patients, p<0.01. The IVRT was significantly prolonged in PSS+ compared to PSS- rTOF children, p<0.05. Furthermore, in PSS+ patients, a significantly lower total deformation in basal lateral segment was observed when compared to rTOF PSS- children. There was no significant difference in QRS duration between PSS+ (139.0±17.4ms) and PSS- (129.3±35.9 ms) children of the study group. Maximal and mean pressure gradient between right ventricular outflow tract and main pulmonary artery did not differ significantly in the PSS+ and PSS- patients and were respectively in PSS+ 23.0±12.6 mmHg and 12.3±7.0 mmHg and in PSS- 23.2±14.0 mmHg and 12.4±7.9 mmHg. Severe pulmonary regurgitation was seen in 12 (44.4%) PSS+ and in 5 (20%) PSS- patients but this difference was not statistically significant. All 3 patients with severe tricuspid regurgitation were PSS+. There were no significant differences between any of the remaining parameters of right ventricular function for PSS+ and PSS- patients. Table 4.

**Table 4:** Echocardiography in PSS+ and PSS—patients.

Parameter		PSS+, mean±SD	PSS-, mean±SD	p
RV ESA/BSA		14.3±6.8	13.6±5.7	NS
RV EDA/BSA		20.0±8.2	18.9±7.6	NS
RV EDA/BSA		20.0±8.2	18.9±7.6	NS
TAPSE [mm]		16.5±3.8	16.5±3.9	NS
RVFAC [%]		28.7±8.6	27.9.4±7.2	NS
Systolic function	S’ [cm/s]	5.2±1.7	6.2±1.3	NS
	IVCT [ms]	121.4±58.3	112.2±60.5	NS
	BL ε[%]	-27.7±8.6	-37.8±12.2	**<0.01**
	ML ε[%]	-21.3±8.3	-25.3±9.1	NS
	AL ε[%]	-14.5±7.6	-19.3±7.2	NS
Diastolic function	E [cm/s]	92.8±11.5	90.1±19.0	NS
	A [cm/s]	64.2±13.1	71.2±18.3	NS
	Edt [ms]	185.7±57.6	196.2±42.5	NS
	E’[cm/s]	4.8±1.8	8.4±2.6	**<0.01**
	A’[cm/s]	3.5±1.5	4.1±1.5	NS
	IVRT[ms]	95.9±35.9	77.0±35.0	**<0.05**
	E/E’	21.7±7.5	12.3±6.7	**<0.01**
	E/A	1.5±0.3	1.3±0.4	NS

PSS+ post systolic shortening present, PSS- post systolic shortening absent, max PG—maximal systolic pressure gradient between right ventricular outflow tract and pulmonary artery, mean PG- mean systolic pressure gradient between right ventricular outflow tract and pulmonary artery, TAPSE–transannular plane systolic excursion, RV FAC–right ventricular fractional area change, RV ESA/BSA–right ventricular end systolic area indexed to body surface area, RV EDA/BSA right ventricular end diastolic area indexed to body surface area, E—maximal early tricuspid inflow velocity, A—atrial tricuspid inflow velocity, Edt–E deceleration time, E/A–E/A ratio, S’–peak systolic velocity of the tricuspid valve, IVCT—isovolumic contraction time, E’—peak early diastolic velocity, A’ peak late diastolic velocity, IVRT- isovolumic relaxation time, E/E’—E/E’ ratio.

### Patients and controls PSS+

In PSS+ rTOF children, the mean filling time was 324.5±101.7 ms, the PVCT was 379.2±44.1 ms. No significant differences in the mean durations of the cardiac cycle between PSS+ patients and controls were found, where the mean PVCP was 354.7±26.2 ms and PVCT 356.5±27.6 ms. The mean TTP level in the BL for the study group was 452.5±53.0 ms whilst that for healthy children was significantly less at 365.0±29.3 ms (p<0.01).

The PSST and PSSTi, in the basal lateral segment were both significantly higher in children after rTOF compared to controls; respectively 73.4 ± 29.8 ms and 24.4 ± 11.1% versus 8.5 ± 4.0 ms and 2.3 ± 1.1% (p<0.01). In PSS+ children the mean ε and εPVCT in the BL for the study group were significantly higher than for controls; respectively -27.7±8.6% and -24.6±9.0% versus -40.9±11.5% and -36.8±11.1% (p<0.01). Likewise, in those PSS+, the mean εPSS and εPSSi in a given segment were not significantly different between patients and controls. In rTOF children, such values were found to be respectively -3.0±1.7% and 12.4±8.2% compared to -4.1±1.6% and 11.0±5.6% for healthy children.

### PSST in rTOF patients

In PSST+ rTOF patients, the QRS duration (*r* = 0.44; *P*<0.05), the ESA/BSA (r = 0.44; p<0.05) and EDA/BSA (r = 0.46; p<0.05) correlated positively whilst TAPSE correlated negatively with PSSTi (*r* = -0.48; *p*<0.05). No correlations were observed between PSSTi and other functional parameters such as: RV FAC, maximal and mean systolic pressure gradient between right ventricle and main pulmonary artery.

## Discussion

In children after tetralogy of Fallot surgical repair, progressive right ventricular dysfunction is universally observed, although despite many studies, the underlying pathophysiology is still not fully known. One of the reasons of progressive deterioration of the right ventricular function might be postsystolic shortening phenomenon.

The possible mechanism responsible for the PSS presence is myocardial degeneration (fibrosis) universally driving to right ventricular elevated filling pressure. In our study we found a significant decrease in myocardial early diastolic velocity (E’) and isovolumic relaxation time prolongation (IVRT) within the right ventricle in patients in whom postsystolic shortening was detected when compared with the remaining subjects of the study group. Hitherto, in the literature, there have been no descriptions of diastolic dysfunction when determined by Tissue Doppler Imaging in rTOF patients with postsystolic shortening. Some trials show, prolonged IVRT upon TDI testing, decrease early maximum diastolic velocities (E’) and increases the E/E’ ratio reflects high end-diastolic pressure and/or right ventricular stiffness in selected patient groups. [17–21]. However in our trial increased E/E’ ratio reflects solely E’ decrease. Thus, whether diastolic function impairment in rTOF children with PSS phenomenon is due to increased myocardial stiffness is difficult to distinguish. Such question could be answered by the studies using CTR or histopathology examinations in patients with PSS + and PSS-. A reduced Edt and an elevated A wave in PSS+ patients is another suggestion that the latter might be associated with diastolic RV dysfunction, although in our study statistical significance was not reached between PSS + and PSS- patients.

The presented study for the first time demonstrates some link between PSS and decreased local deformation. We found reduced strain in the basal lateral segment of the right ventricle in those patients for whom postsystolic shortening was detected as compared to the remaining rTOF children.

We suggest that PSS influences local deformation and does not affect global systolic function of the right ventricle. RV FAC was unrelated to PSS in our study. A negative correlation between PSSTi duration and TAPSE, in our circumstances, most likely confirms local contractile dysfunction in the basal lateral segment of the right ventricle where the parameter is typically measured. According to the trials TAPSE does not reflect global systolic function in children after TOF correction [22].

Except the intrinsic myocardial impairment, there are other mechanisms considered to be responsible for the presence of PSS. Hui et al. suggest [13] postsystolic shortening in rTOF patients is an expression of electromechanical dyssynchrony. Indeed in our study QRS duration correlates with PSSTi although there is no difference of QRS duration in rTOF PSS+ and PSS- children.

Nevertheless, we found that PSSTi was significantly correlated to parameters such indexed end-systolic and end-diastolic areas of right ventricle, indicating that the volume overload does also have some link with PSS. On the other hand, the magnitude of pulmonary valve regurgitation has been shown to be less related to PSS; both in our study and work by Hui et al [13]. It thus seems that further studies are required to determine postsystolic shortening changes, in relation to the chronic volume overload observed rTOF children.

Based on our results, right ventricular pressure overload did not affect PSS. There was no difference in the maximum and average systolic pressure gradient within the right ventricular outflow tract between patients with PSS + and PSS-. The systolic pressure gradients within right ventricular outflow tract also poorly correlated with PSST. However, once should note that the values of the pressure gradients were low in our study group, the average value of mean pressure gradient was 12.3 mmHg and only in 2 patients the maximum pressure gradient exceeded of 50 mmHg.

As it was presented in previous studies the time to peak parameter might become extended after the moment when the arterial valve closes in healthy ones [13,23]. Light of these findings, we compared the duration of cardiac cycle phases in patients and healthy children when postsystolic shortening occurred, indicating, that for the basal lateral segment, these durations were significantly longer in rTOF children than in healthy controls. This difference persisted after indexing up to the duration of diastole. The insignificant prolonging of deformation following valve closure can be assumed to be a well-tolerated feature, but if this time is extended beyond a set threshold, does right ventricular function impairment worsen.

Prolonged deformation after aortic valve closure in the left ventricle is fairly well understood. It is of prognostic value in patients with myocardial infarction. Recognizing this symptom in patients suffering from myocardial infarction directly after revascularization, improves the prognosis in the reversion back to normal contractile function. However, when this occurs in patients with arterial hypertension or dilated cardiomyopathy, then it is associated with impaired diastolic function, as detected by Tissue Doppler Imaging or Speckle Tracking Echocardiography [9–12]. In patients with complete left bundle branch block, the septal flash consequently also occurs leading to postsystolic shortening. In such cases, *Parsai et al* [24] found resynchronization to be a highly effective therapy in those patients. The presented findings demonstrate that left ventricular postsystolic shortening on one side is linked with impaired synchronicity of systolic and diastolic function, as well as preserving myocardial viability and reversible lesions.

By extrapolating study data from the left ventricle, it might be assumed that the postsystolic shortening findings obtained from within the right ventricle might provide useful information in qualifying patients for therapeutic interventions following Tetralogy of Fallot correction. However, there is no evidence on whether pulmonary valve replacement, (due to severe regurgitation) or cardiac resynchronization, could significantly improve mechanical function of the disturbed right ventricle due to postsystolic shortening in rTOF patients.

### Limitations

The lack of data about long-term clinical implications is a major limitation of our study. Besides, measuring the pulmonary pulse wave Doppler profile and 2D images for strain were not simultaneous, but possibly heart rate variability might influence postsystolic shortening time. We therefore performed the measurement only if the heart rate in PW and 2D 4 chamber views were similar (maximal accepted difference of RR intervals in ECG was 5%) as described previously in the literature. We also calculated the postsystolic shortening index to avoid any risk of inadequately assessing postsystolic shortening connected with heart rate changes. We did not assess right ventricular systolic pressure because of insufficient tricuspidal regurgitation, so pressure overloading was based only on the gradient between the right ventricle and the main pulmonary artery.

We also consider that at present there are many questions that remain unanswered (eg. How PSS impacts on the extent of fibrosis in the RV myocardium? How PSS quantitatively affects regurgitation of the pulmonary and tricuspid valves?) MRI, histopathology and exercise studies might provide the answers, which were not performed on our subjects. The follow-up of PSS assessment could also explain the issue of by how much PSS is an indicator of long-term myocardial damage.

## Conclusions

1. Tissue Doppler Echocardiography and Speckle Tracking Echocardiography are useful techniques for detecting regional systolic and diastolic dysfunction in children after Tetralogy of Fallot surgical repair.
2. Postsystolic shortening in the basal lateral segment is commonly seen in children after the Tetralogy of Fallot surgical repair, and is associated with altered right ventricular systolic and diastolic function.

## Supporting Information

S1 Fig Correlation between postsystolic shortening time index and right ventricular end-diastolic area derived from apical four chamber view indexed to body surface area. (PDF)

S2 Fig Correlation between postsystolic shortening time index and right ventricular end-systolic area derived from apical four chamber view indexed to body surface area. (PDF)

S3 Fig Correlation between postsystolic shortening time index and QRS duration in the ECG. (PDF)

S4 Fig Correlation between postsystolic shortening time index and tranannular plane systolic excursion. (PDF)
