# Supplementary material for: Postsystolic Shortening Is Associated with Altered Right Ventricular Function in Children after Tetralogy of Fallot Surgical Repair
Source: PLoS One. 2017 Jan 3;12(1):e0169178. doi: 10.1371/journal.pone.0169178 (PMC5207703; doi:10.1371/journal.pone.0169178)

Correlation between postsystolic shortening time index (PSSTi) and right ventricular end-diastolic area derived from apical four chamber view indexed to body surface area (EDA/BSA).

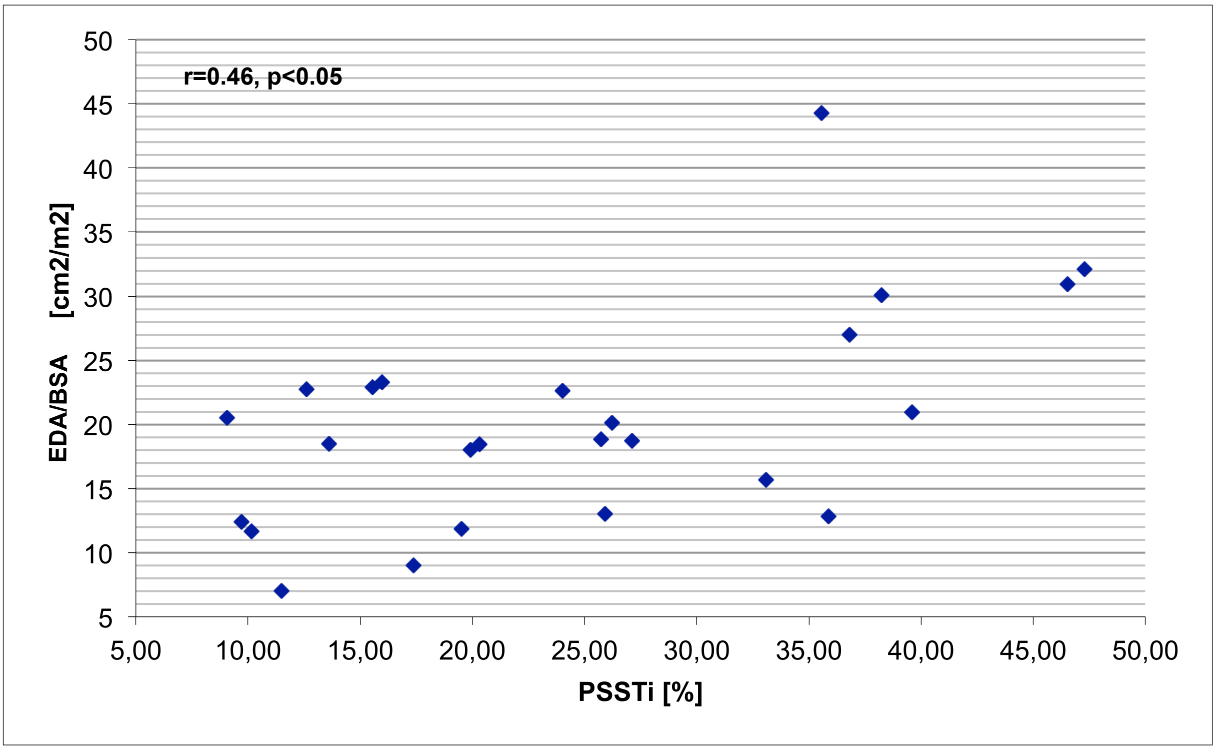

Supplement: S1 Fig — (PDF) [file pone.0169178.s001.pdf]
