# Supplementary material for: Postsystolic Shortening Is Associated with Altered Right Ventricular Function in Children after Tetralogy of Fallot Surgical Repair
Source: PLoS One. 2017 Jan 3;12(1):e0169178. doi: 10.1371/journal.pone.0169178 (PMC5207703; doi:10.1371/journal.pone.0169178)

Correlation between postsystolic shortening time index and right ventricular end-systolic area derived from apical four chamber view indexed to body surface area.

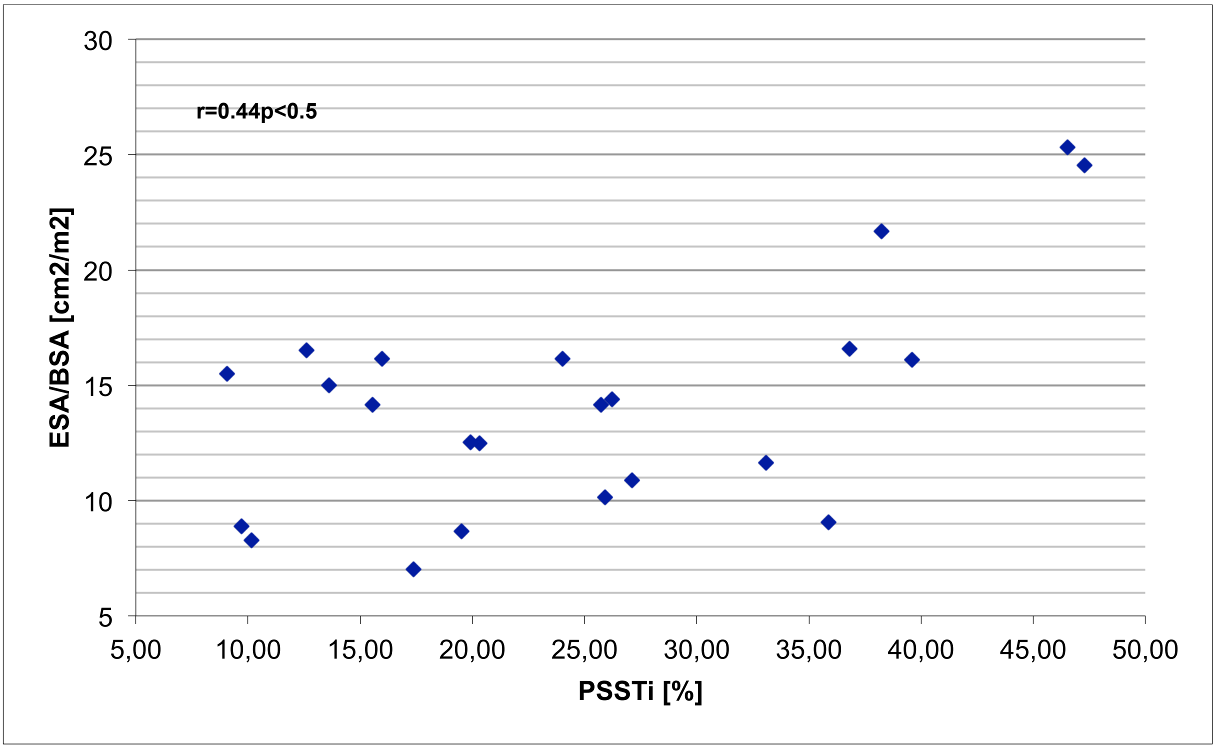

Supplement: S2 Fig — (PDF) [file pone.0169178.s002.pdf]
